# A Leaf Disc Assay for Evaluating the Response of Tea (*Camellia sinensis*) to PEG-Induced Osmotic Stress and Protective Effects of Azoxystrobin against Drought

**DOI:** 10.3390/plants10030546

**Published:** 2021-03-13

**Authors:** Yu-Chieh Chiu, Bo-Jen Chen, Yen-Shuo Su, Wen-Dar Huang, Chang-Chang Chen

**Affiliations:** 1 Department of Agronomy, National Taiwan University, Daan, Taipei 101, Taiwan; r07621105@ntu.edu.tw; 2 Tea Research and Extension Station, New Taipei 22391, Taiwan; bojen329@ttes.gov.tw (B.-J.C.); tres561@ttes.gov.tw (Y.-S.S.); 3 National Research Institute of Chinese Medicine, Ministry of Health and Welfare, Beitou, Taipei 112, Taiwan

**Keywords:** *Camellia sinensis*, leaf discs, osmotic stress, azoxystrobin, chlorophyll fluorescence

## Abstract

Tea (*Camellia sinensis*), a globally cultivated beverage crop, is sensitive to drought, which can have an adverse effect on the yield and quality of tea. Azoxystrobin (AZ) is one kind of fungicide considered as an agent to relieve damage caused by stress. Initially, the response of tea plant to osmotic-gradient stress was evaluated using leaf disc assays with PEG-induced osmotic stress. The decline of the maximum quantum yield of PSII (F_v_/F_m_), actual photosynthetic efficiency of PS II (Y(II)), total chlorophylls, carotenoids, DPPH radical scavenging capacity, reducing power, total phenols, and the increase in MDA was observed in leaf discs treated with a gradient of PEG solutions (22.8, 33.2, 41.1% PEG, and blank). These results revealed that efficiency of photosystem II (PSII), photosynthetic pigments, and antioxidant ability in leaf discs were inhibited with an aggravated lipid peroxidation under PEG-induced osmotic stress, and indicated leaf disc assay with moderate PEG iso-osmotic condition would reflect a portion of tea plant response to drought stress. Therefore, the protective effect of AZ (0.125 and 1.25 g a.i. L^−1^) on tea plants suffering from drought was evaluated using leaf disc assays with 22.8% PEG iso-osmotic condition. Pretreatment of AZ (0.125 a.i. g L^−1^) reversed F_v_/F_m_, Y(II), DPPH radical scavenging capacity, and reducing power with reduced MDA in PEG-treated leaf discs, but photosynthetic pigments, total phenols, and ascorbate peroxidase activity were irresponsive to AZ. An Alleviated physiological damage in tea leaf with AZ applying was preliminarily revealed in this study. A Rapid screening of agents for tea plants against drought was developed to assist in the selection of protective agents.

## 1. Introduction

Tea (*Camellia sinensis*) is an important beverage crop with expanding plantation areas and boosting production in the world [1]. Worldwide tea production had reached 6.50 million tons on 5.08 million hectares in 2019 [2]. Tea plants are cultivated in a wide range of climatic environments, although tea plants prefer a warm and humid climate. In Taiwan, tea is the most important beverage crop, and *C. sinensis* var. *sinensis* is the major cultivated tea variety that contributed to over 90% of tea production. The tea (*C. sinensis* var. *sinensis*) cultivar “Taiwan Tea Experiment Station No.12 (TTES No.12)”, which is also called “Jhinhsuan”, is one of the most widely grown tea cultivars in Taiwan. The traits of TTES No.12, including growth vigor, resistance to diseases and insect pests, and yield, are better than other landraces in Taiwan, and its vertical type canopy is more suitable for mechanical harvesting [3]. Areas receiving less than 1250 mm rainfall per year are inadequate for industrial production, thus major nations of tea cultivation are distributed in tropical and subtropical areas [1]. Drought stress leads to the physiological, biochemical, and morphological responses and transcriptional regulation in tea plants, and can cause a decline in the quality and yield of tea leaves [4,5]. A decline in soil moisture made leaves shrivel temporarily and then caused plants to wither in the absence of immediate irrigation [6]. The occurrence of seasonal drought every half year was insufficient for tea production in main tea producing regions of East Africa, such as Tanzania and Malawi [7]. Moreover, recent climate change resulted in a decrease in rainfall during monsoon season in India and Sri-Lanka severely impacted the tea industry within the Indian subcontinent [4,8]. Taiwan is also located in a climate impact hotspot, with the impact of drought on crop production [9]. Drought led to oxidative stress, resulting from the accumulation of reactive oxygen species (ROS), and then caused lipid peroxidation, enzyme denaturation, chlorophyll degradation, and photosynthetic apparatus dysfunction [10,11]. Therefore, there is an urgent need to maintain growth and to promote resistance to drought in the tea plant.

Application of exogenous chemicals, including melatonin, abscisic acid [12], and/or microelements [13], such as calcium (Ca), potassium (K), manganese (Mn), zinc (Zn), boron (B) [14], can relieve drought-induced damages and enhance drought-resistance and/or restorative growth in tea plants. Many studies have reported that strobilurins, systemic and broad-spectrum fungicides for control crop diseases, also provide physiological and growth benefits in plants that were not infected by fungus, such as antioxidant and anti-aging, increase in nitrogen assimilation, photosynthesis, and yield [15,16]. Likewise, azoxystrobin (AZ), one kind of strobilurin, provided a similar function in crop production [17,18,19,20]. Some studies illustrated that the application of AZ improved nitrogen use and physiological traits in photosynthesis, antioxidant status, and delaying senescence in wheat and decelerated chlorophyll degradation in harvested lettuce [20,21,22,23]. Moreover, applying AZ-enhanced water use, root architecture, and yield in tomatoes during drought stress. AZ treatment also prevented leaf senescence, enhanced photosynthetic physiology and yield in wheat under water deficit [21,24,25]. Additionally, AZ was shown to have low toxicity to mammals and a lower residue in harvested food [26]. AZ is also one recommended fungicide in controlling tea plant diseases in Taiwan. However, the effect or benefit of AZ in tea plants against drought stress was rarely evaluated.

Chlorophyll fluorescence analysis is a rapid and non-destructive method to monitor the level of stress-induced damage in plants [27], and has been applied to survey drought resistance and/or drought induced damage in numerous crops, including the tea plant [28,29,30]. Recently, prompt chlorophyll a fluorescence and related parameters derived from OJIP transient curves were responsive to multiple stress in plants [31,32,33,34]. Similarly, delayed chlorophyll a fluorescence, based on the reemission signal from PSII antenna complex, demonstrates that the complex phase was also used in screening photosynthetic response under stress [35]. The moderate integration of non-destructive approaches, including gaseous exchange and chlorophyll fluorescence, can effectively be used to screen photosynthetic response in plants to drought and/or salt stress [36,37]. A decrease in the maximum quantum yield of PSII (F_v_/F_m_) reflects the photosynthetic potential in plants under stress, which might damage the membrane system in photosynthetic organelles in plant tissue, block electron transport in photosystem II (PSII), and result in photoinhibition [38]. Effective quantum yield of PSII (Y(II)), quantum yield of regulated energy dissipation of PSII (Y(NPQ)), and quantum yield of non-regulated energy dissipation of PSII (Y(NO)) reflect photochemical reaction, photoprotection, and photodamage, respectively [6,39]. Consequently, chlorophyll fluorescence parameters were used to evaluate the response to drought stress in this study.

Polyethylene glycol (PEG) is a water-soluble polymer that does not penetrate roots, thus it is widely used to induce osmotic stress. The germination of wheat under PEG-induced drought [40], the germination and seedling responses of desert plants to PEG-induced drought [41], and the physiological response of rapeseed leaf to PEG-induced osmotic stress have been demonstrated previously [42,43]. Both physiological (leaf age, root distribution, plant nutrient, and health status of the tea plant) and environmental factors (temperature, humility, strength of wind, and soil moisture), and their interactions influence leaf water potential [44,45]. Water potential in tea leaves is maintained around −0.2 and −0.4 MPa under most climatic conditions, although some cultivars maintain water potential at −1.0 MPa. However, during dry periods water potential can drop to −4.9 MPa [8,13]. PEG iso-osmotic solution can be used to closely simulate drought level to evaluate the response of tea leaves to drought.

In this study, we hypothesized that AZ might provide a protective effect on tea plants. However, tea plants are a perennial brush, and a diverse responses of tea plants to drought were observed in our previous investigation (data not shown). Therefore, leaf disc assay, which is considered an effective tool for rapid evaluation of physiological responses in plants, is an alternative approach to survey preliminary information [42,43,46]. The objectives of this presented study were to determine a daily physiological response of tea to a gradient of PEG-induced osmotic stress by leaf disc assay initially, and then to evaluate the effect of exogenous AZ on tea leaf physiology under moderate PEG-induced drought conditions.

## 2. Results

### 2.1. Chlorophyll Fluorescence in Leaf Discs Collected from PEG-Induced Osmotic Stress

**Figure 1:**
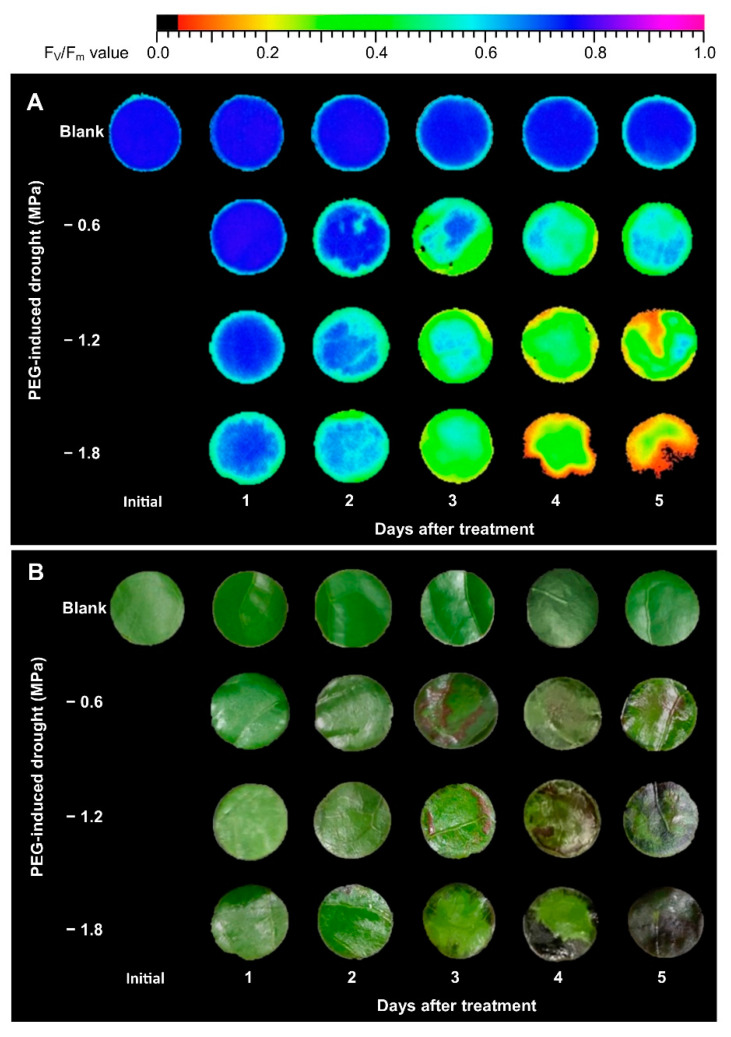
Chlorophyll fluorescence image (**A**) and leaf appearance correspond to F_v_/F_m_ (**B**) value under PEG-induced osmotic stress during a 5-day period.

**Figure 2:**
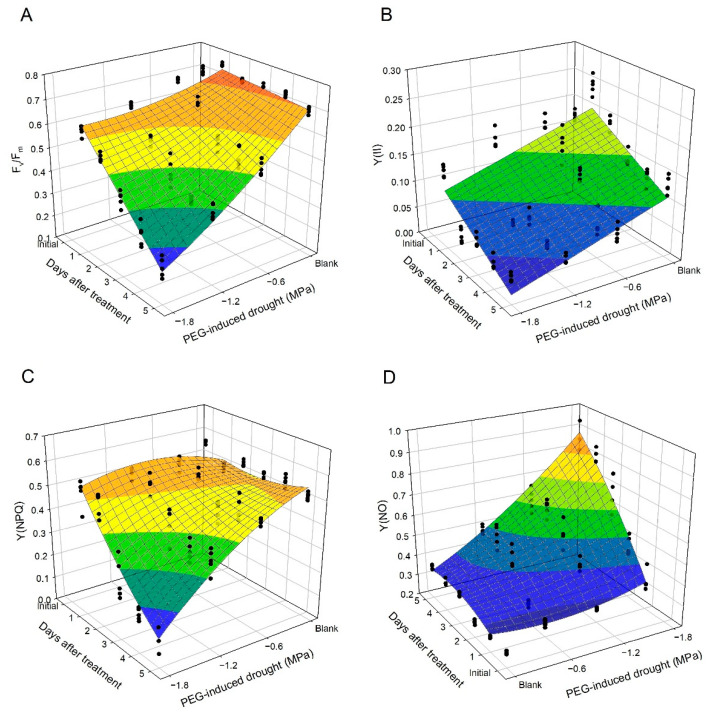
Scatter plots and regression surfaces of the maximum quantum yield of PSII (F_v_/F_m_) (**A**)_,_ actual photosynthetic efficiency of PS II (Y(II)) (**B**), quantum yield of regulated energy dissipation (Y(NPQ)) (**C**), and quantum yield of non-regulated energy dissipation (Y(NO)) (**D**) value in tea leaf discs as affected by PEG-induced osmotic stress during a 5-day period.

Components of chlorophyll fluorescence can be used to evaluate the status of photosystem II (PSII) in plant tissue. The response of F_v_/F_m_ (determined after dark-adaptation) in leaf discs collected from PEG-induced osmotic stress for a period of 5 days is presented in Figure 1A and Figure 2A. F_v_/F_m_ values of untreated leaf discs ranged from 0.75 to 0.80 over a 5-day period. However, F_v_/F_m_ of PEG-treated leaf discs declined with drought gradient and with the increasing number of days after treatment. According to the regression model for F_v_/F_m_ (R^2^ = 0.936), both linear and quadratic effects of PEG-induced (d and d^2^), linear effect of the number of days after treatment (t), and interaction effects (dt) were significant (Table 1). The appearance of PEG-treated leaf discs also showed necrosis after the third day of treatment (Figure 1B).

Y(II), Y(NO), and Y(NPQ) in leaf discs collected from the PEG-induced experiment were determined at 185 μmol m^−2^ s^−1^ of photosynthetically active radiation (PAR). The initial value of Y(II) in untreated leaf discs was 0.17, while Y(II) was also declined with drought gradient and days after treatment (Figure 2B). The linear effect of drought and days (d and t) were both significant in the regression model for Y(II) (R^2^ = 0.715) (Table 1). The initial value of Y(NPQ) was 0.56, while Y(NPQ) decreased suddenly under severe drought (−1.2 and −1.8 MPa) from the third to the fifth day (Figure 2C). According to the model for Y(NPQ) (R^2^ = 0.828), all of d, d^2^, t, t^2^, and dt effects were significant (Table 1). The initial value of Y(NO) in untreated leaf discs was 0.27 (Figure 2D), while an increase in Y(NO) with increases in drought gradient and the number of days after treatment was observed. In the regression model for Y(NO) (R^2^ = 0.914), the linear and quadratic effect of drought and days (d, d^2^, t, and t^2^) and interaction effect (dt) were significant (Table 1).

### 2.2. Photosynthetic Pigments in Leaf Discs Collected from PEG-Induced Osmotic Stress

**Figure 3:**
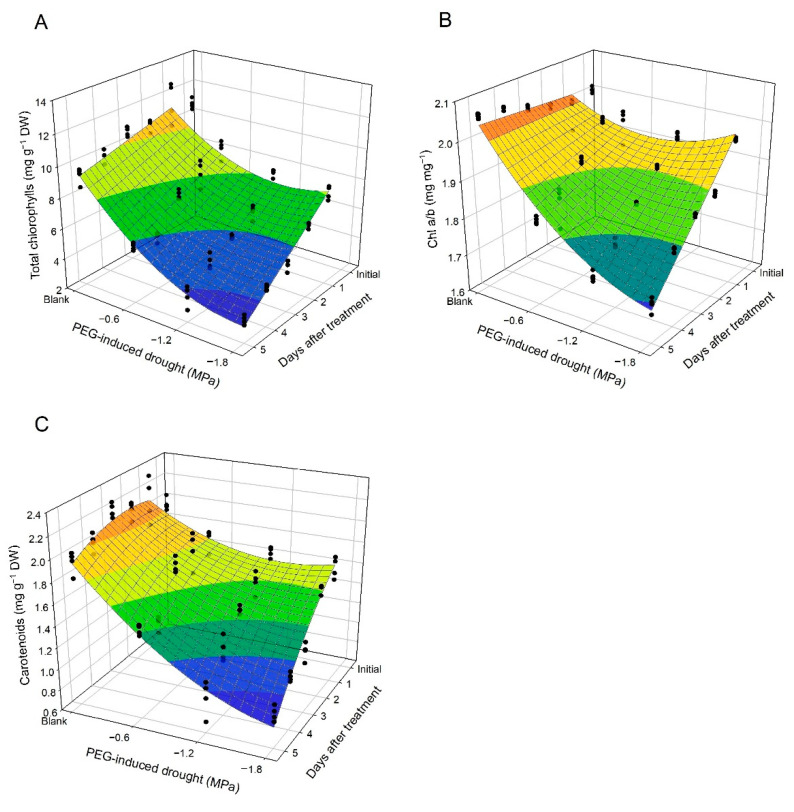
Scatter plots and regression surfaces of total chlorophyll (**A**), Chl *a*/*b* ratio (**B**), and carotenoids (**C**) in tea leaf discs as affected by PEG-induced osmotic stress during a 5-day period.

The initial total chlorophylls (Chl) in untreated leaf discs was 10.74 mg g^−1^ DW, and then Chl in untreated leaf discs declined slightly from the first to the fifth day. However, Chl in PEG-treated leaf discs felled with drought gradient and days after treatment (Figure 3A). The effects of d, d^2^, and t, and were significant in the regression model for Chl (R^2^ = 0.940) (Table 1). Chl a/b in untreated leaf discs remained stable at 1.97–2.06 mg mg^−1^, while Chl a/b in PEG-treated leaf discs diminished with drought gradient and number of days after treatment (Figure 3B). In the model for Chl a/b (R^2^ = 0.902), effects of d, d^2^, and dt were significant, whereas t effect was insignificant (Table 1). Carotenoids (Car) in untreated leaf discs was scattered from 1.94 to 2.18 mg g^−1^ DW during the 5-day period, while content of Car in PEG-treated leaf discs dropped with drought gradient and number of days after treatment (Figure 3C). All effects of d, d^2^, t, t^2^, and dt were significant in the model for Car (R^2^ = 0.909) (Table 1).

### 2.3. Antioxidant Status in Leaf Discs Collected from PEG-Induced Osmotic Stress

**Figure 4:**
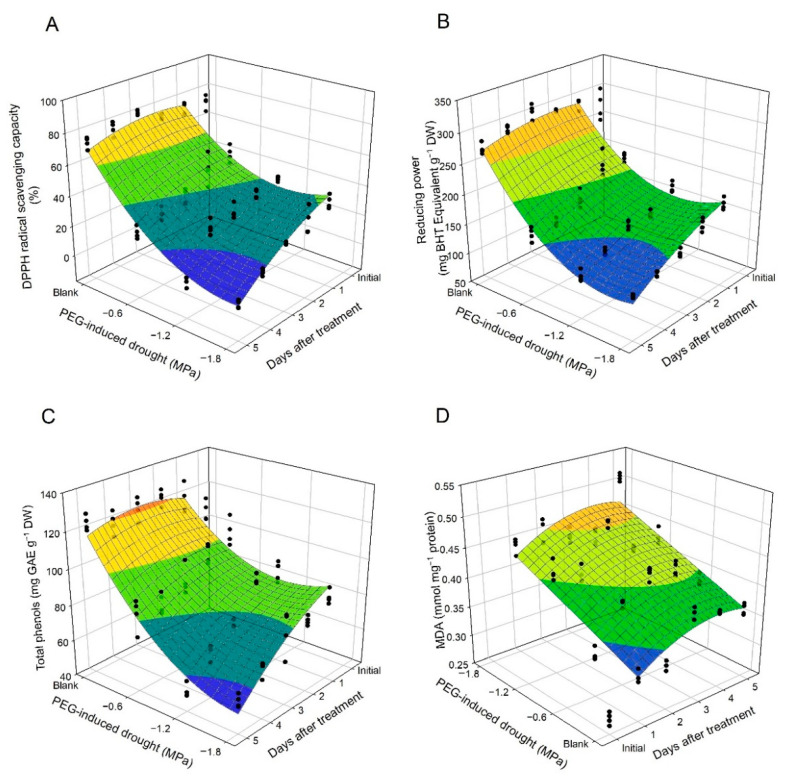
Scatter plots and regression surfaces of DPPH radical scavenging capacity (**A**), reducing power (**B**), total phenols (**C**), and MDA (**D**) content in tea leaf discs as affected by PEG-induced osmotic stress during a 5-day period.

Antioxidant status was evaluated using assays of 1,1-diphenyl-2-picryl-hydrazyl (DPPH) radical scavenging capacity, reducing power, and content of total phenols in this study. DPPH radical scavenging capacity in untreated leaf discs ranged from 71.21 to 80.52%, while a decrease in DPPH radical scavenging capacity in PEG-treated leaf discs with drought gradient and number of days after treatment was observed (Figure 4A). All effects were significant, whereas effects of d and d^2^ were more major than others according to estimated coefficient in the model for DPPH radical scavenging capacity (R^2^ = 0.915) (Table 1). Similar trends were observed in scatter plots of reducing power and total phenols in leaf discs (Figure 4B,C). All effects in the model for total phenols were significant (R^2^ = 0.883), whereas the t effect was insignificant in the model for reducing power (R^2^ = 0.942) (Table 1). The MDA is considered to be an index of lipid peroxidation resulting from instability of cell membrane caused by stress-induced ROS [47]. MDA in untreated leaf discs increased slightly from 0.28 to 0.39, while MDA in PEG-treated leaf discs suddenly increased under drought gradient within 3 days (Figure 4D). Effects of d, t, and t^2^ were significant in the model for MDA (R^2^ = 0.743) (Table 1).

### 2.4. Chlorophyll Fluorescence in Leaf Discs of Exogenous AZ Experiment

**Figure 5:**
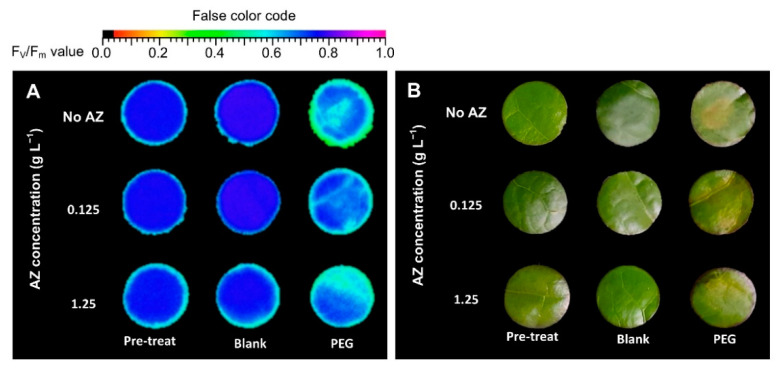
Chlorophyll fluorescence image (**A**) and leaf appearance correspond to F_v_/F_m_ (**B**) value of tea leaf discs with pre-treatment (Pre-treat) of three different azoxystrobin (AZ) levels and then treated by blank solution (Blank) and PEG-induced osmotic stress (PEG), respectively.

**Figure 6:**
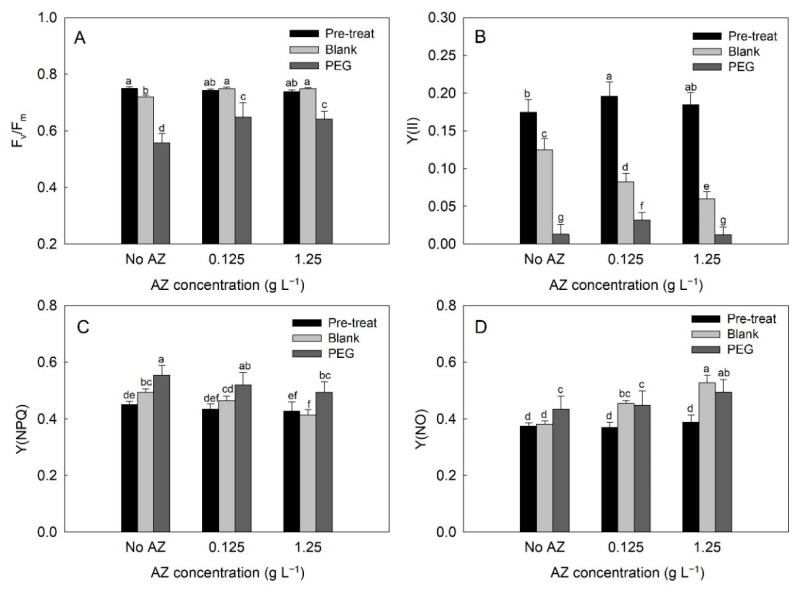
Change in the maximum quantum yield of PSII (F_v_/F_m_) (**A**)_,_ actual photosynthetic efficiency of PS II (Y(II)) (**B**), quantum yield of regulated energy dissipation (Y(NPQ)) (**C**), and quantum yield of non-regulated energy dissipation (Y(NO)) (**D**) value of tea leaf discs with pre-treatment (Pre-treat) of three different azoxystrobin (AZ) levels and then treated by blank solution (Blank) and PEG-induced osmotic stress (PEG), respectively. Values are the means (*n* = 5) with standard errors shown by vertical bars. Different letters (a–g) represent statistically different means (LSD, *p* < 0.05).

F_v_/F_m_ in leaf discs that were pre-treated with 3 levels of exogenous AZ for 1-day ranged from 0.74 to 0.75, and then a lower F_v_/F_m_ was observed in leaf discs without AZ (no AZ) under no PEG (blank) for 3 days. Meanwhile, F_v_/F_m_ in leaf discs with exogenous AZ stayed at 0.75. F_v_/F_m_ in leaf discs without AZ (no AZ) under PEG-induced osmotic stress (PEG) dropped dramatically, and exogenous AZ prevented a sudden reduction in F_v_/F_m_ in leaf discs under PEG treatment (Figure 5A and Figure 6A). The diverse response of F_v_/F_m_ in leaf discs of the exogenous AZ experiment were difficult to detect based on appearance (Figure 5B).

Y(II) in leaf discs with 0.125 g AZ L^−1^ (0.20) was significantly higher (*p* < 0.05) than without AZ (0.17) after a 1-day pre-treatment, while Y(II) under the blank treatment declined with increasing exogenous AZ on the third day after treatment. A sharp decrease in Y(II) under PEG treatment was observed on the third day after treatment, and Y(II) with 0.125 g AZ L^−1^ pre-treatment was higher than the others (Figure 6B). Y(NPQ) in leaf discs after 1-day pre-treatment was 0.43–0.45. Under blank treatment for 3 days, Y(NPQ) declined with an increase in exogenous AZ. A similar trend was observed in Y(NPQ) under PEG-induced osmotic stress (Figure 6C). Y(NO) in leaf discs after 1-day pre-treatment was 0.37–0.39. Under blank treatment for 3 days, Y(NO) increased with an increase in exogenous AZ. Furthermore, Y(NO) in leaf discs treated with 1.25 g AZ L^−1^ was significantly higher (*p* < 0.05) than others under PEG-induced osmotic stress 3 days after treatment (Figure 6D).

### 2.5. Photosynthetic Pigments in Leaf Discs of Exogenous AZ Experiment

The dynamics of Chl, Chl a/b, and Car in leaf discs in the exogenous AZ experiment are listed in Table 2. Chl of leaf discs without AZ (no AZ) was 9.87 mg g^−1^ DW after the 1-day pretreatment and remained at 9.90 mg g^−1^ DW under the blank treatment for 3 days, while a reduction in Chl was observed in leaf discs under PEG-induced osmotic stress on the third day after treatment. Similar trends were observed in Chl in AZ pre-treated leaf discs. Overall, Chl in leaf discs with 1.25 g AZ L^−1^ pre-treatment was significantly lower (*p* < 0.05) than no AZ added and 0.125 g AZ L^−1^ pre-treatments. Chl a/b in leaf discs without AZ (no AZ) was 1.883 mg mg^−1^ after 1-day pre-treatment, and raised to 1.912 mg mg^−1^ under blank for 3 days, while a significant reduction (*p* < 0.05) of Chl a/b was observed under PEG-induced osmotic stress for 3 days. A similar trend was also found in Chl a/b in AZ pre-treated leaf discs, but lower Chl a/b was observed in leaf discs with 1.25 g AZ L^−1^. Car in leaf discs declined with increasing AZ concentration after the 1-day pretreatment. Car in leaf discs without AZ (no AZ) increased from 1.71 to 1.95 and 1.97 mg g^−1^ DW under the blank and PEG-induced osmotic stress treatments, respectively. Under blank treatment, an increase in Car in leaf discs was also observed. However, exogenous AZ did not promote Car in leaf discs under PEG-induced osmotic stress.

### 2.6. Antioxidant Status in Leaf Discs of Exogenous AZ Experiment

**Figure 7:**
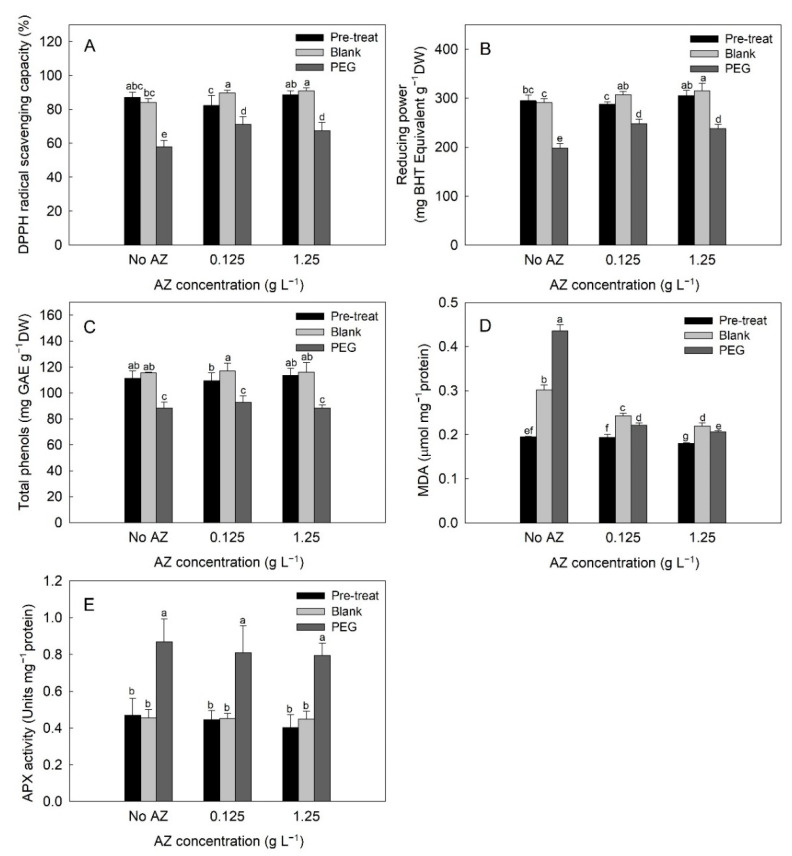
Change in DPPH radical scavenging capacity (**A**), reducing power (**B**), total phenols (**C**), malondialdehyde (MDA) (**D**), and ascorbate peroxidase (APX) activity (**E**) of tea leaf discs with pre-treatment (Pre-treat) of three different azoxystrobin (AZ) levels and then treated with blank solution (Blank) and PEG-induced osmotic stress (PEG), respectively. Values are the means (*n* = 4) with standard errors shown by vertical bars. Different letters (a–g) represent statistically different means (LSD, *p* < 0.05).

DPPH radical scavenging capacity in leaf discs after 1-day pretreatment was 82.29–88.48%. Under blank treatment for 3 days, this capacity in leaf discs with exogenous AZ was significantly higher (*p* < 0.05) than the no-added treatment. Furthermore, DPPH radical scavenging capacity was significantly reduced (*p* < 0.05) under PEG-induced osmotic stress, while exogenous AZ enhanced this capacity during drought (Figure 7A). A similar trend was observed in the reducing power in leaf discs of exogenous AZ experiment (Figure 7B).

Total phenol in leaf discs after 1-day pretreatment was 109.3–114 mg GAE g^−1^ DW. A significant increase in total phenol (*p* < 0.05) in 0.125 g AZ L^−1^ pre-treated leaf discs was observed under blank treatment for 3 days. Meanwhile, total phenol was reduced significantly (*p* < 0.05) under PEG-induced osmotic stress, and the differences among 3 levels of exogenous AZ were insignificant (Figure 7C). MDA in leaf discs after 1-day pretreatment decreased slightly from 0.195 to 0.180 μmol mg^−1^ protein with increase in AZ concentration. A sudden increase in MDA in leaf discs was observed under blank and PEG-induced osmotic stress for 3 days, whereas exogenous AZ significantly (*p* < 0.05) inhibited MDA under both treatments (Figure 7D). Ascorbate peroxidase (APX) activity in leaf discs was additionally determined in the exogenous AZ experiment. Among all AZ levels, APX activity in leaf discs was 0.40–0.47 units mg^−1^ protein after 1-day pretreatment and blank treatment for 3 days. Nonetheless, APX activity under PEG-induced osmotic stress increased by a factor of 2, regardless of the exogenous AZ level (Figure 7E).

## 3. Discussion

Drought causes several disadvantages to crops, such as causing early aging, reducing yield, and inhibiting photosynthesis. It was known that morphological responses of tea plant to drought included deep root systems and smaller but thicker leaves with more numerous stomata, responses of physiology and biochemistry in tea plant included inhibited photosynthetic physiological traits (such as declined net CO_2_ assimilation, reduced photochemical quenching, and induced non-photochemical quenching) with a decrease in chlorophylls and carotenoids, an accumulation of osmolytes (proline, glycine betaine, and soluble sugars), and lower activity of enzymatic antioxidant systems (APX, catalase, glutathione reductase, and peroxidase) with a reduction in non-enzymatic antioxidants (phenolic, ascorbate, and glutathione) and an increase in ROS and MDA, and transcriptional levels of genes involved in mentioned morphological, physiological, and biochemical responses to drought were regulated [5].

Photosynthetic inhibition under drought conditions blocks the electron transport chain in chloroplasts and the accumulation of ROS damages the photosynthetic apparatus and leads to lipid peroxidation and enzyme denaturation [48]. To create a drought gradient, a PEG-induced simulation of osmotic stress at the leaf level provides more exact environmental control, enhanced reproducibility, and a shorter experiment duration than experiments performed at the whole-plant level [42]. The tea plant is a perennial woody crop, thus PEG simulation with a leaf disc assay avoids the effects of soil moisture or other ambient environmental factors in the tea garden, and individual differences in each tea plant [8]. According to our preliminary data of F_v_/F_m_ in leaf discs under PEG gradient (data not shown), the osmotic levels included in this study were −0.6 MPa (22.8% PEG), −1.2 MPa (33.2% PEG), and −1.8 MPa (41.1% PEG).

Chlorophyll fluorescence is considered to be a rapid screening test for the evaluation of photosynthesis in plants under drought stress [27]. F_v_/F_m_ is one of the chlorophyll fluorescence parameters and is measured after dark adaptation. Previous studies reported relatively low F_v_/F_m_ values in plants accompanied by severe osmotic stress [49,50]. In this study, F_v_/F_m_ in non-treated leaf discs (blank) was more stable over a 5-day period than PEG-treated leaf discs (Figure 1A and Figure 2A). According to the regression model, the decrease in F_v_/F_m_ in leaf discs of the blank group, which declined 0.008 per day, was a more gradual decline than that of the PEG-treated group (Table 1). Y(II), Y(NO), and Y(NPQ) are three parameters of chlorophyll fluorescence characterizing the energy allocation of the reaction center in PSII [47]. Y(II) reflects the photochemical reactions in PSII under illumination. Y(NPQ) is considered to be a useful parameter for evaluating excessive energy dissipated via non-photochemical mechanisms, such as the xanthophyll cycle, D1 repair cycle, and photorespiration, whereas Y(NO) is an important parameter in evaluating the level of photo-damage, which is quantified in proportion to the excessive energy dissipated via heat [50]. In this study, reduced Y(II) and Y(NPQ), but enhanced Y(NO), in the PEG-treated leaf discs (Figure 2B–D and Table 1) suggested that PEG-induced osmotic stress resulted in photo-inhibition in the leaves and the excessive light energy was dissipated via heat (which is harmful to photosynthetic tissue), rather than photochemical or non-photochemical dissipation in PSII [47,50]. The response of chlorophyll fluorescence to the drought-induced damage to photosynthesis in the tea plant and other plants has been reported previously [28,47].

The degradation of chlorophylls and carotenoids is one process of stress-induced senescence in plants, and it is attributed to the degradative reaction center complexes and light-harvesting complex in PSII, resulting in the loss of function of PSII and photosynthetic activity [51]. The photosynthetic pigment results of this study suggest that total chlorophylls, Chl a/b, and carotenoids declined with decreasing osmotic potential (Figure 3 and Table 1). Netto et al. [52] also revealed that PEG-induced osmotic stress led to the degradation of photosynthetic pigments in the tea plant. F_v_/F_m_ did not drop as sharply as total chlorophylls in the leaf discs of the blank group over a 5-day period (Figure 2A and Figure 3A, and Table 1), perhaps due to stable Chl a/b (Figure 3B and Table 1). Dinç et al. [53] demonstrated that F_v_/F_m_ was irresponsive to the dynamics of photosynthetic pigments while Chl a/b was constant.

Phenolic compounds, in correlation with tea quality, can scavenge ROS to reduce oxidative damage in tea leaves; therefore, total phenols in tea are considered to be one of parameters promoting drought tolerance in tea plant [54]. The antioxidant status in PEG-treated leaf discs over 5 days (Figure 4 and Table 1) showed that osmotic stress resulted in a decrease in phenolic compounds, DPPH radical scavenging capacity, and reducing power in tea leaf, with an increasing MDA under osmotic stress. These results were similar to our previous study of wheat [55]. MDA assay is one assessment of lipid peroxidation which is degraded from cellular membrane systems attacked by stress-induced ROS in correlation with leaf senescence [20,47]. The described dynamics of chlorophyll fluorescence, photosynthetic pigments, and antioxidant status in this study (Figure 1, Figure 2, Figure 3 and Figure 4 and Table 1) were a portion of tea plant responses to drought stress, but these results were consistent with mentioned previously [5]. Therefore, leaf disc assay with moderate PEG iso-osmotic condition would be a rapid and alternative tool to screen the protective effect of chemicals and/or natural products on tea plant against stress for a preliminary evaluation in this study.

AZ is a fungicide for control crop diseases and is also considered as a growth regulator that would recover CO_2_ assimilation rate and stomatal conductance [21]. Additionally, the application of AZ increased water use efficacy, cell membrane stability, enzymatic antioxidant systems (catalase, peroxidase, polyphenol oxidase), total phenols, root growth to improve drought tolerance, and fruit yield in tomato [24,25]. Several previous studies indicated that exogenous AZ can enhance chlorophyll content, promote antioxidative ability to prevent cellular oxidative status, alleviating stress damage [20,22]. The benefits of exogenous AZ to chlorophyll fluorescence in leaves under stress remained controversial [16]. Our previous study revealed that exogenous AZ inhibited photochemical and photo-protection parameters and led to severe photodamage in wheat seedlings under heat stress [39]. Barányiová and Klem [21] also indicated that the application of AZ during drought was unconducive to chlorophyll fluorescence because of reduced stomatal conductance and blocked gas exchange in wheat that was not infected by fungus. The recommended concentration of AZ application in tea gardens is 0.125 g L^−1^ with a 21-day pre-harvest interval in Taiwan, therefore 0.125 and 1.25 AZ g AZ L^−1^ were applied in this study. Furthermore, in accordance with the drought-induced response of chlorophyll fluorescence in leaf discs (Figure 2), the effect of exogenous AZ on leaf discs was evaluated under −0.6 MPa (22.8% PEG) on the third day following treatment in this study.

In comparison with the no AZ added group, F_v_/F_m_ in PEG-treated leaf discs was enhanced at 0.125 and 1.25 AZ g L^−1^ (Figure 5A and Figure 6A), but only Y(II) was elevated in PEG-treated leaf discs 0.125 g AZ L^−1^ (Figure 6B). As speculated by Dinç et al. [53], the promoted photochemical reaction in PEG-treated leaf discs with 0.125 g AZ L^−1^ might be attributed to constant Chl a/b (Table 2), whereas a lower Y(NPQ) and a higher Y(NO) in PEG-treated leaf discs at 1.25 g AZ L^−1^ (Figure 6C,D) suggests that a higher concentration AZ did not benefit photo-protection but resulted in severe photo-damage. The lower photochemical reaction in leaf discs at high concentration AZ implies that tea leaves have to dissipate excessive light energy via regulated and non-regulated mechanisms. More severe photo-damage with less photo-protection in leaf discs at high concentrations of AZ also indicates that tea leaves may suffer serious oxidative stress under these conditions [21]. Moreover, declines in total Chl and Chl a/b in PEG-treated leaf discs, both with and without AZ (Table 2), suggests that AZ did not influence chlorophylls in this study. Nevertheless, an increase in chlorophylls with AZ application has been published previously [23,39]. However, other studies reported that exogenous AZ did not promote photosynthetic pigments in drought-stressed wheat [21] or rice plants that were not infected by fungus [56].

Exogenous AZ would alleviate oxidative stress and stabilize cellular membrane systems in plants under drought conditions [20]. In accordance with the result of PEG-treated leaf discs with AZ pretreatment in this study, exogenous AZ was not effective on phenolic compounds, but benefited the DPPH radical scavenging capacity and reducing power, with lower MDA in tea leaves against drought (Figure 7A,B,D). On the other hand, APX activity was irresponsive to exogenous AZ treatment (Figure 7E), consistent with our previous findings [39]. AZ is involved with the regulation of antioxidant enzymes, with the exception of APX activity [15], and mitigation of yield loss caused by fungal disease [57] has been demonstrated previously. As speculated by Debona and Rodrigues [15], the protective effect of AZ should be attributed to reducing oxidative stress through control of fungal infection.

The benefit and/or protective effect of AZ on tea plants were rarely studied previously. In this study, we have preliminarily evaluated a portion of the protective effect of AZ on chlorophyll fluorescence and antioxidant status in tea plant with a leaf disc assay. However, a comprehensive evaluation of the positive effect of AZ is necessary at the whole-plant level and/or in field condition because of the inherent limitation of leaf disc assay.

## 4. Materials and Methods

### 4.1. Plant Materials and Treatments

The tea plant (*C. sinensis* var. *sinensis*) cultivar ‘TTES No.12′ was used in this study. New flushes were obtained from tea plants in the experimental tea gardens of Wenshan Substation of Tea Research and Extension Station, New Taipei City, Taiwan (24.95° E, 121.63° N) on 16 October 2019 and 3 March 2020, respectively. Chlorophyll content in the flushes, determined by using a SPAD-502 chlorophyll meter (Konica Minolta, Tokyo, Japan), ranged from 55 to 65 SPAD value were selected to prepare leaf discs with a cork borer (12-mm inner diameter).

A total of 20 leaf discs were uniformly placed into covered Petri dishes (60 mm diameter) containing 5 mL of treatment solution, with the abaxial surface exposed. For the drought gradient experiment, leaf discs collected on 16 October 2019 were treated with the osmotic agent, polyethylene glycol (PEG-6000, Sigma-Aldrich), for 1–5 day(s), respectively. According to the method of Michel and Kaufmann [58], PEG was used to prepare and simulate three levels of drought, −0.6 MPa (22.8% (*w*/*v*) PEG), −1.2 MPa (33.2 % PEG), and −1.8 MPa (41.1% PEG). A solution without PEG was considered as the blank. The drought gradient experiment was independently performed four times for a randomized design of incubation. For the exogenous AZ experiment, leaf discs collected on March 3, 2020 were pre-treated with 5 mL of AZ fungicide (250 g a.i. L^−1^, Amistar^®^, Syngenta Limited, Waterford, Ireland) at concentration of 0.125, 1.25 g AZ L^−1^, and blank solution without AZ (No AZ) for 1 day, and then solution in dish was replaced by 5 mL of 22.8% (*w*/*v*) PEG or blank solution for 3 days, respectively. The exogenous AZ experiment was independently performed six times for a randomized design of incubation. Each dish was well sealed by Parafilm and incubated in growth chambers at 24 °C and photosynthetic photon flux density (PPFD) was uniformly set at 300 μmol m^−2^ s^−1^ with 24 h light. The chlorophyll fluorescence of treated leaf discs was determined before sample collection.

### 4.2. Measurements of Chlorophyll Fluorescence

Treated leaf discs were dark-adapted for 30 min before measurements. Chlorophyll fluorescence was measured and averaged in the center portion (1154 pixel disc) of each leaf disc taken at ambient temperature with a Chl fluorometer imaging-PAM (Walz, Effeltrich, Germany). Saturating light and actinic light intensities were set to 3700 and 185 μmol m^−2^ s^−1^ of PAR, respectively. F_v_/F_m_, Y(II), NPQ, Y(NPQ), and Y(NO) were determined according to previously described methods [59].

### 4.3. Determination of Photosynthetic Pigments

The method of Chl and Car measurement in leaf discs followed, with modification, the study of Yang et al. [60]. Briefly, 0.01 g of lyophilized sample powder was extracted with 5.4 mL 80% (*v*/*v*) acetone solution, and then centrifuged at 12,400 rpm and 4 °C for 10 min. The supernatant of the sample extract was collected. The concentrations of Chl a, Chl b, and carotenoids in the sample solution was determined using a spectrophotometer (SpectraMax^®^ ABS Plus) at 663.6, 646.6, and 440.5 nm. Total Chl and Chl a/b was calculated by sum of Chl a and Chl b and their ratio, respectively.

### 4.4. Estimation of Total Phenols

The method of Singleton et al. [61] was followed with slight modification to estimate the total phenols in treated leaf discs, with gallic acid as a standard phenolic compound. Briefly, 0.01 g of lyophilized sample powder was extracted with 5 mL of 60% (*v*/*v*) methanol solution with 0.3% (*v*/*v*) HCl, and then centrifuged at 12,400 rpm and 4 °C for 10 min. Then, 2 mL of 1N Folin–Ciocalteu reagent and 2 mL of 10% (*w*/*v*) sodium carbonate solution were added to the sample solution and incubated for 2.5 h. The total phenols of supernatant was determined by using a spectrophotometer (SpectraMax^®^ ABS Plus) at 750 nm.

### 4.5. Antioxidant Capacity Assays

0.01 g of lyophilized sample powder was ultrasonically extracted with 1.8 mL methanol for 30 min and left overnight at 4 °C. Next day, the extract was centrifuged at 12,400 rpm and 4 °C for 10 min. Then, 450 μL of the supernatant was collected and diluted with 800 μL of methanol. The diluted extract was stored at −20 °C for antioxidant capacity assays.

The six-fold diluted extract was prepared for reducing power assay, which was analyzed according to the method of Oyaiz [62] with butylated hydroxytoluene (BHT) as a standard. The four-fold diluted extract was prepared for DPPH radical scavenging capacity assay, which was measured according to the method of Blois [63] with Butylated hydroxytoluene (BHT) as a standard.

### 4.6. Determination of Lipid Peroxidation

Lipid peroxidation in leaf discs was determined by malondialdehyde (MDA) measurement. The method of Heath and Packer [64] was modified to determine the MDA concentration. Briefly, 0.02 g of lyophilized sample powder was ground and extracted with 1 mL of 5% (*w*/*v*) trichloroacetic acid (TCA), and then centrifuged at 12,400 rpm and 4 °C for 10 min. Then, 500 μL of supernatant was collected and mixed with 2 mL of 0.5% (*w*/*v*) thiobarbituric acid (TBA), which was made up with 20% TCA. The mixture was incubated at 95 °C for 30 min with a water bath, and then immediately cooled and degassed in an ice bath for 10 min. The reaction mixture was centrifuged at 12,400 rpm and 4 °C for 10 min. The MDA concentration of sample mixtures was determined by using a spectrophotometer (SpectraMax^®^ ABS Plus) at 532 and 600 nm. A blank was also determined.

### 4.7. Measurement of Ascorbate Peroxidase Activity

The method of Nakano and Asada [65] was followed with slight modification to measure the ascorbate peroxidase (APX) activity in leaf discs collected from the AZ experiment. Briefly, 2 leaf discs were ground and extracted with 1 mL of 50 mM sodium phosphate buffer (pH 6.8) in an ice bath, and then centrifuged at 4 °C and 12,000 rpm for 20 min. Then, 0.1 mL of supernatant was collected and mixed with 1 mL of 150 mM potassium phosphate buffer (pH 7.0), 0.4 mL of 0.75 mM ethylenediaminetetraacetic acid (EDTA), 1.5 mL of 1.5 mM ascorbate, and 0.5 mL of 6 mM H_2_O_2_. The mixture was collected used to measure the absorbance at 290 nm with a spectrophotometer (Hitachi U3010, Tokyo, Japan), and APX activity was evaluated based on the change in absorbance at 290 nm.

### 4.8. Statistical Analysis

The drought experiment data were subjected to statistical analysis through multiple polynomial regression analysis using the REG procedure of SAS 9.3 software (Cary, NC, USA). The general model of multiple polynomial regression with interaction effects is presented as the following,

y_ij_ = β_0_ + β_1_ d_i_ + β_2_ d_i_^2^ + β_2_ t_j_ + β_4_ t_j_^2^ + β_5_ d_i_ t_j_ + ε_ij_ (1)

where y was a dependent variable, d and d^2^ were linear and quadratic effects of PEG-induced osmotic stress, respectively, t and t^2^ were linear and quadratic effects of the number of days after treatment, respectively, dt was interaction effect, β_0_ was intercept, β_1_–β_5_ were regression coefficients, and ε was error. The insignificant quadratic and/or interaction factors in the polynomial regression were removed, and the process was continued step-by-step to the point at which there were no insignificant quadratic and/or interaction factor(s) remaining.

All data in the AZ experiment were subjected to statistical analysis by using analysis of variance (ANOVA), followed by a least significant difference (LSD) test at *p* < 0.05. All statistical analyses were conducted using the GLM procedure of SAS 9.3 software.

## 5. Conclusions

In this study, the responses of chlorophyll fluorescence, photosynthetic pigments, lipid peroxidation, total phenols, and antioxidant ability in leaf discs to a gradient of PEG iso-osmotic condition illustrated a portion of drought-related response in tea plant and suggested leaf disc assay with moderate PEG iso-osmotic condition would be an alternative approach to screen the protective effect of chemicals against drought. Furthermore, the effect of AZ on PEG-induced changes of tea leaf in PEG iso-osmotic condition was preliminarily demonstrated in the presented research. Application of exogenous AZ moderately enhanced antioxidant ability and alleviated lipid peroxidation in PEG-treated leaf discs, whereas photosynthetic pigments and chlorophyll fluorescence were irresponsive to exogenous AZ. The positive effect and protective mechanism of AZ against drought in the tea plant warrant further investigation at the whole-plant level under drought stress and/or field conditions due to the inherent limitation of leaf disc assay. Additionally, a rapid screening of a plant protection agent against drought in tea plant was developed in this study. This screening technique can be used to identify and select chemicals or natural products for further field experimentation to identify novel agents that are useful in promoting drought resistance in the tea plant.

## Figures and Tables

**Table 1:** Multiple regression models for F_v_/F_m_, Y(II), Y(NO), Y(NPQ), total chlorophyll (Chl), Chl a/b, Car, 1,1-diphenyl-2-picryl-hydrazyl (DPPH) radical scavenging capacity, MDA, total phenols, reducing power, and their root-mean-square error (root MSE), R^2^, and adjusted R^2^ (adj-R^2^).

Effect	Regression Coefficient										
	F_v_/F_m_	Y(II)	Y(NO)	Y(NPQ)	Total Chl	Chl a/b	Car	DPPH Scavenging	Reducing Power	Total Phenols	MDA
Intercept	0.740 ***	0.223 ***	0.229 ***	0.538 ***	11.513 ***	2.009 ***	2.008 ***	69.911 ***	281.493 ***	112.184 ***	0.285 ***
d	0.059 **	0.051 ***	0.137 ***	−0.204 ***	4.468 ***	0.097 ***	0.494 ***	50.697 ***	163.475 ***	42.046 ***	−0.054 ***
D^2^	0.033 **	-	0.053 ***	−0.069 ***	1.848 ***	0.077***	0.229 ***	20.964 ***	66.495 ***	18.553 ***	-
t	−0.008 *	−0.023 ***	0.067 ***	−0.033 *	−0.452 ***	0.005 ^NS^	0.098 **	5.409 *	8.513 ^NS^	7.081 **	0.044 ***
t^2^	-	-	−0.009 ***	0.007 **	-	-	−0.023 ***	−1.173 **	−2.238 **	−1.241 **	−0.006 ***
dt	0.051 ***	-	−0.069 ***	0.063 ***	0.456 ***	0.047 ***	0.108 ***	4.390 ***	8.642 ***	5.547 ***	-
Root MSE	0.037	0.033	0.051	0.052	0.613	0.034	0.119	6.815	14.879	7.774	0.028
R^2^	0.936	0.715	0.914	0.828	0.940	0.902	0.909	0.915	0.942	0.883	0.743
Adj-R^2^	0.933	0.710	0.910	0.819	0.937	0.897	0.904	0.909	0.938	0.876	0.733

d, linear effect of PEG-induced osmotic stress; d^2^, quadratic effect of PEG-induced osmotic stress; t, linear effect of days after treatment; t^2^, quadratic effect of days after treatment; dt, interaction effect. ^NS^, not significant; *, *p* < 0.05; **p < 0.01; ***, *p* < 0.001.

**Table 2:** Change in total chlorophylls (Chl), Chl *a*/*b*, and carotenoids (Car) in tea leaf discs with pre-treatment (Pre-treat) of three different azoxystrobin (AZ) levels followed by treatment with a blank solution (Blank) and PEG-induced osmotic stress (PEG), respectively.

AZ Conc. (g L^−1^)	Treatment	Total Chl (mg g^−1^ DW)	Chl a/b (mg mg^−1^)	Car (mg g^−1^ DW)
No AZ	Pre-treat	9.87 ± 0.43 a	1.883 ± 0.005 cd	1.71 ± 0.07 cd
	Blank	9.90 ± 0.38 a	1.912 ± 0.011 ab	1.95 ± 0.07 a
	PEG	8.90 ± 0.35 cd	1.807 ± 0.016 f	1.97 ± 0.07 a
0.125	Pre-treat	9.45 ± 0.41 ab	1.880 ± 0.012 de	1.63 ± 0.05 d
	Blank	9.80 ± 0.31 a	1.926 ± 0.007 a	1.85 ± 0.08 b
	PEG	8.43 ± 0.25 d	1.790 ± 0.020 f	1.68 ± 0.03 cd
1.25	Pre-treat	8.84 ± 0.38 cd	1.861 ± 0.004 e	1.54 ± 0.07 e
	Blank	9.16 ± 0.47 bc	1.900 ± 0.011 bc	1.74 ± 0.08 c
	PEG	7.60 ± 0.21 e	1.761 ± 0.021 g	1.51 ± 0.05 e

Within columns, means ± SE (*n* = 4) followed by the same letter are not significantly different, according to LSD (*p* < 0.05).

## Data Availability

The data presented in this study are available from the authors.
